# Results after skin traction for femur shaft fractures in children below the age of four years

**DOI:** 10.1007/s00068-022-01996-x

**Published:** 2022-05-31

**Authors:** Markus Dietzel, Leon Ole Schöneberg, Matthias Schunn, Simon Scherer, Michael Esser, Hans Joachim Kirschner, Jörg Fuchs, Justus Lieber

**Affiliations:** 1 Department of Pediatric Surgery and Pediatric Urology, University Children’s Hospital, Tübingen, Germany; 2 Department of Diagnostic Radiology, University Hospital, Hoppe-Seyler-Str. 3, 72076 Tübingen, Germany

**Keywords:** Pediatric trauma, Pediatric femur fracture, Skin traction, Overhead extension, Leg-length discrepancy

## Abstract

**Purpose:**

Nonsurgical management has been identified as the treatment of choice for femoral shaft fractures in children below four years of age. For various reasons, the surgical approach has become increasingly popular in recent years. The aim of this study is to report results after vertical skin traction and analyze the benefits of this technique as well as to point out advantages compared with surgery in this age group.

**Methods:**

The authors performed a retrospective data analysis, including all patients with femoral shaft fractures below the age of four who were treated with vertical skin traction at our institution between January 2006 and December 2016.

**Results:**

Skin traction for a femoral shaft fracture was performed for 36 patients (mean age 1.6 years; 1 day–3.5 years). The mean duration of traction was 18.5 days (14–30). Complications included soft tissue affections (*n *= 5), which all healed spontaneously. Consolidation was observed in all fractures. Initial axial deviations and shortening did not change during traction until consolidation (*p* > 0.05), and no relevant torsion deformity occurred (*p* = 0.01). Patients gained full weight-bearing within 12.3 days (7–40) following end of traction. At the final follow-up, after a mean of 29.3 months (12–192), leg-length discrepancy (mean 7.1 mm; 5–20) was found on radiograms in nine cases, and axial deviations (mean 7.7°; 5–25) were documented in seven cases. None of the patients had limitations in daily activities or sports.

**Conclusion:**

Skin traction is a technically easy, safe, and non-invasive treatment modality for femoral shaft fractures in children below the age of four years. Strong results are obtained benefited from a high potential of growth-related correction, and in principle no anesthesia is needed. A prolonged hospitalization and socio-economic factors maintain the ongoing debate in comparison with the surgical approach.

**Level of evidence:**

Level III, retrospective.

## Introduction

Fractures of the femoral shaft account for 0.7–1.7% of all pediatric fractures and 3.5% of long-bone extremity fractures in children [1, 2]. These fractures occur in every age group with an average age at injury of 6.3 years old, and injury mechanisms are mostly age-dependent, ranging from trivial falls at home to high-velocity accidents [3, 4]. In general, the treatment for femoral shaft fractures depends on factors, such as the age, size, and weight of the patient, fracture type, soft tissue integrity, concurrent injuries, family preference, and surgeon preference [5, 6]. For younger children (below 4–5 years old), predominantly conservative management is described, and satisfactory results have been obtained throughout [1, 5, 7, 8]. Some authors note that even though the overwhelming majority of pediatric patients with femoral shaft fractures are expected to heal with normal function and radiographic alignment, minor sequelae of leg-length discrepancy, torsion and angular deformity, and back pain are often described [9, 10]. Other authors state that angulation and overlap or shortening tend to correct with remodeling, especially in younger children [7]. However, there is an increasing shift toward surgical therapy [11], which can be explained by various socio-economic factors [12–15]. Nevertheless, there currently is no consensus on the best method of managing femoral shaft fractures in younger children.

The aim of this study is to analyze the implementation and results after skin traction for femoral shaft fractures in children below four years of age at a single-center institution.

## Materials and methods

### Patients and ethical considerations

This study retrospectively analyzed all patients below the age of four undergoing skin traction for femoral shaft fractures at our institution between January 2006 and December 2016. The study was approved by the local ethical committee (011/2018BO2). Data on demographic characteristics, fracture type, and extent of dislocation, implementation of skin traction, hospital stay, complications, and outcomes were all collected from hospital records and stored on a computerized database. Fractures were classified according to the AO Pediatric Comprehensive Classification of Long-Bone Fractures [16]. Data were acquired and processed according to the latest version of the World Medical Association Declaration of Helsinki—Ethical Principles for Medical Research Involving Human Subjects.

**Fig. 1:**
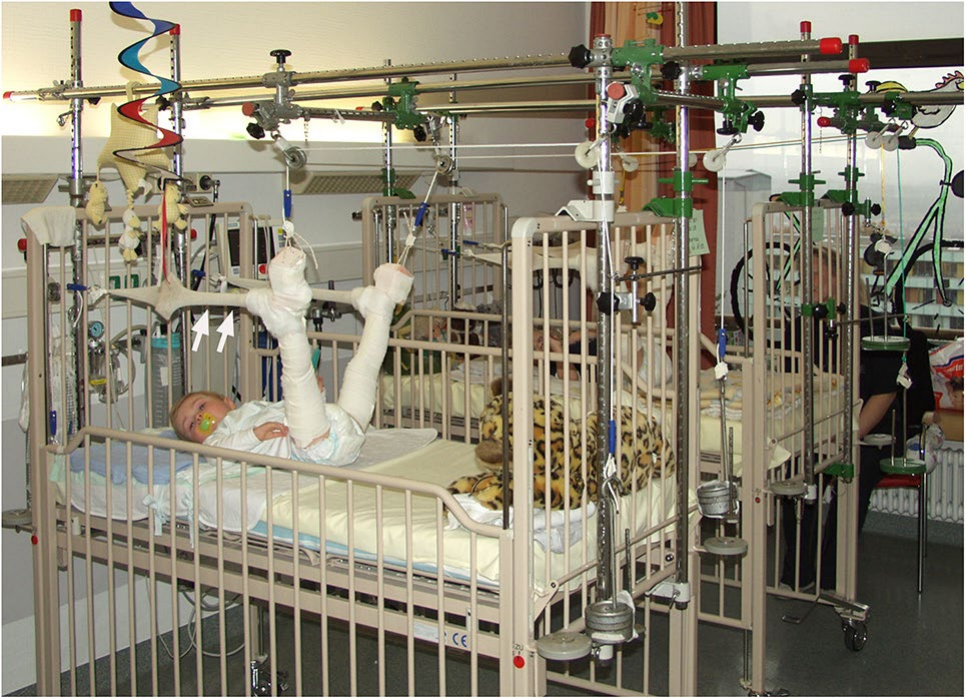
A vertical overhead skin traction device installed to a hospital bed for the treatment of femoral shaft fractures in two infants. A weight with 1/6 of the patient’s bodyweight was fixed to each leg via a spring-loaded pulley off the side of the bed. To prevent malrotation, the bed stretcher was equipped with additional slings (↑↑) pulling headwards

Vertical skin traction was performed according to the department's protocol in children below the age of three years or with a bodyweight below 15 kg. The duration of traction was three weeks, while it was two weeks for birth-related fractures. Therapy was performed with a traction device attached to a hospital bed. In all patients, a weight with 1/6 of the patient’s bodyweight was fixed to each leg via a spring-loaded pulley off the side of the bed. To prevent malrotation, the bed stretcher was equipped with additional slings pulling headwards (Fig. 1). After 48–72 h of traction, when children are adapted and relaxed, X-rays in two planes of the femur were performed. Eventually either the direction of traction or the weight was modified to optimize alignment. All patients received daily conscientious observation for soft tissue erosions, tension blisters, and an unimpaired peripheral circulation, motor function, and sensitivity. At the end of treatment, an X-ray was performed to ensure consolidation. After discharge, all patients were encouraged to regain their mobility individually under the guidance of their parents. Follow-up in the outpatient clinic was continued for at least 12 months and until no restrictions were reported. Each patient was examined for abnormalities in gait, leg-length discrepancies, and torsion and angular deformities of the affected limb. Conventional X-rays were performed to document the growth-related correction of angular deformities; standing roentgenograms were used to determine leg-length discrepancies. The extent of fracture displacement at trauma, at consolidation, and at the last follow-up were documented and statistically compared.

### Statistics

Statistical analysis was performed using Student’s *t* tests (SPSS Statistics, IBM, Armonk, NY, USA). All *p* values < 0.05 were considered statistically significant.

## Results

For 36 patients (mean age 1.6 years; 1 day–3.5 years) between January 2006 and December 2016, skin traction for 30 spiral/oblique (AO 32-D/5.1) and 6 transvers femoral shaft fractures (32-D/4.1) were performed. Table 1 shows demographic and clinical data of all patients. Traction was applied in analgosedation (*n *= 18), after the administration of peripheral analgesics (*n *= 14), or in general anesthesia (*n *= 4). General anesthesia was chosen if the treating surgeon aimed to perform a reduction or if analgosedation was not sufficient. The mean duration of traction was 18.5 days (14–30). Complications included soft tissue affections (*n *= 5), which all healed spontaneously. Consolidation was observed in all fractures. Comparing the extent of dislocation at the time point of trauma on conventional X-rays with the time point of consolidation, there was no difference (Table 2). This applies to all axial deviations as well as leg shortenings. Only clinically evident torsion deformities at the time point of trauma were corrected through vertical traction and could no longer be detected at the time point of consolidation (*p* = 0.01).

**Table 1:** Demographic and clinical data of 36 patients who underwent skin traction for a femoral shaft fracture at the institution from January 2006 to December 2016

Total patients (*n*)	36	
Male: female (*n*)	27: 9	
Mean age (years)	1.6 (1 day–3.5 years)	
Injury cause (*n*)		
Home accident	18	
Fall during sports or from relevant height	11	
Birth-related trauma	3	
High-energy trauma	2	
Pathological fracture	1	
Child abuse	1	
Fracture type (*n*)		
Oblique/spiral	30	
Transverse	6	
Traction application (*n*)		
Intravenous analgosedation	18	
Peripheral analgesics	14	
General anesthesia	4	
Duration of traction (days)	18.5 (14–30)	
Complications (*n*)		
Tension blisters	3	
Superficial skin maceration	2	
Time to full weight-bearing following end of traction (days)	12.3 (7–40)	
Follow-up (months)	29.3 (12–192)	
Gait disturbances (*n*)	5	
Leg-length discrepancy (clinical)	3	9.6 mm (5–20)
Leg-length discrepancy (radiographic)	9	7.1 mm (4–10)
Axial deviation (radiographic)	7	7.7° (5–25)
Insole support	3

**Table 2:** The extent of fracture displacement in 36 patients with a fracture of the femoral shaft at the time point of injury compared with the extent of fracture displacement at the time point of consolidation and termination of skin traction

	Trauma		Consolidation		*p*
nondisplaced fracture (*n*)	4		5		
Displaced fracture (*n*)	32		31		
Shortening (n/extent)	27	9.8 mm (1–25)	24	9.1 mm (1–15)	0.32
Axial deviation (*n*/extent)	31		28		
Varus	14	13.4° (2–40)	24	12.7° (2–30)	0.47
Valgus	6	6.3° (2–13	2	4.5° (4–5)	0.42
Antecurvation	9	19.7° (2–55)	9	17.2° (3–42)	0.36
Recurvation	8	19.7° (2–40)	8	19.7° (2–24)	0.34
Rotation	4		0		0.01

*p* < 0.05 was considered statistically significant

After discharge, patients received follow-up care in the outpatient clinic. Newborns (*n *= 6) and patients with comorbidities (*n *= 3) were excluded from selected examinations. The remaining patients gained full weight-bearing within 12.3 days (7–40). At the last follow-up, after a mean of 29.3 months (12–192), leg-length discrepancy (mean 7.1 mm; 4–10) was found on X-rays in nine cases. Upon clinical examination, leg-length discrepancy (mean 9.6 mm; 5–20) was found only in three cases. These patients had temporary insole support to offer leg-length compensation until no more complaints were mentioned. Axial deviations measured through X-rays (mean 7.7°; 5–25) were documented in seven cases. At the last follow-up, no restrictions on daily activities or limitations during sports were reported.

## Discussion

The main finding of this study is that skin traction for femoral shaft fractures in young children below the age of four years is an easy, non-invasive, and safe treatment option with satisfactory results and no significant complications. In principle, treatment methods for pediatric femoral shaft fractures depend on various factors, including the age and weight of the patient in particular in small children [1]. Traditionally, conservative treatment plays a predominant role in young children, and spica hip cast and traction methods are available for this purpose [7]. In developing countries, vertical traction is performed in 87.7% of cases, and results are described as good as in Western countries [17]. However, the surgical management of femoral shaft fractures in patients below the age of four years has progressively increased in the past. This trend has been observed in many industrial countries, such as the United Kingdom [18], the United States [11], and Sweden [19]. A Cochrane database analysis reports a 35% increase in surgical therapy and a 58% increase in the group of four to five years of age [20]. In Germany, Strohm et al. have shown that 50% of all patients in the age group of below three years of age with femoral shaft fractures are nowadays treated operatively with elastic stable intramedullary nailing (ESIN). This is done despite the national guidelines’ recommendation of conservative treatment [21]. Since its development, ESIN has been established a gold standard for pediatric long-bone fractures, including the femoral shaft [22]. Given the well-described advantages of this technique, indications for ESIN have constantly extended also to selected age groups [14, 21]. Studies have shown that children whose femoral shaft fractures were treated with ESIN achieved their milestones significantly faster than those children whose fractures were treated with traction and a cast [14]. However, the possible disadvantages of ESIN for younger children continue to be highlighted. For instance, the implantation of a titanium elastic nail close to the highly potent long-lasting distal femoral growth plate may cause growth disturbances. In addition, the trumpet-like configuration of the supracondylar area of the distal femur in younger children may cause longitudinal instability with retrograde ESIN in spiral or oblique fractures. Even though end caps or locking systems may compensate for instability, there is no proof of earlier mobilization with surgery as compared with conservatively treated patients.

The major challenge and drawback of skin traction is the unduly long hospitalization. In our study, patients were hospitalized for a mean of 18.5 days, which is comparable with findings in the literature [14]. In studies reporting an older mean age, the length of hospital stay may exceed six weeks [17]. Associated injuries, comorbidities, and time to learn ambulation non-weight-bearing with crutches are factors that influence the length of hospitalization significantly. In our collective, the mean age was 1.6 years, enabling rapid consolidation. However, there are methods that shorten hospital stay, such as home traction or a short period of traction followed by a hip spica cast [15, 23, 24]. These options should be encouraged in our setting, even though these concepts are associated with other challenges, such as structural requirements, parental acceptance, and the need for a high-quality plastering technique.

Financial aspects have also assumed increased importance in health care. Several investigators have reported the charges associated with various forms of femur fracture treatment. Newton et al. report that the lowest charges are for spica casting. Both skin traction and home traction were associated with significant savings over in-hospital skeleton traction and intramedullary nails [12]. Hedin et al. report that the highest charges are for skin traction, while the lowest are for external fixation [25]. Lewis et al. report that the highest charges are for ESIN and the lowest are for spica casting [26]. However, all studies have concluded that the main factor for determining the cost of treatment was the number of days in the hospital. Nonetheless, none of the studies performed their analysis depending on specific age groups, and none of the studies included possible or actual complications nor additional costs for metal removal in surgically treated patients. Therefore, it remains unknown whether there is a difference in the total charges for the treatment of femoral shaft fractures in children below the age of four years as compared with surgical approaches.

**Fig. 2:**
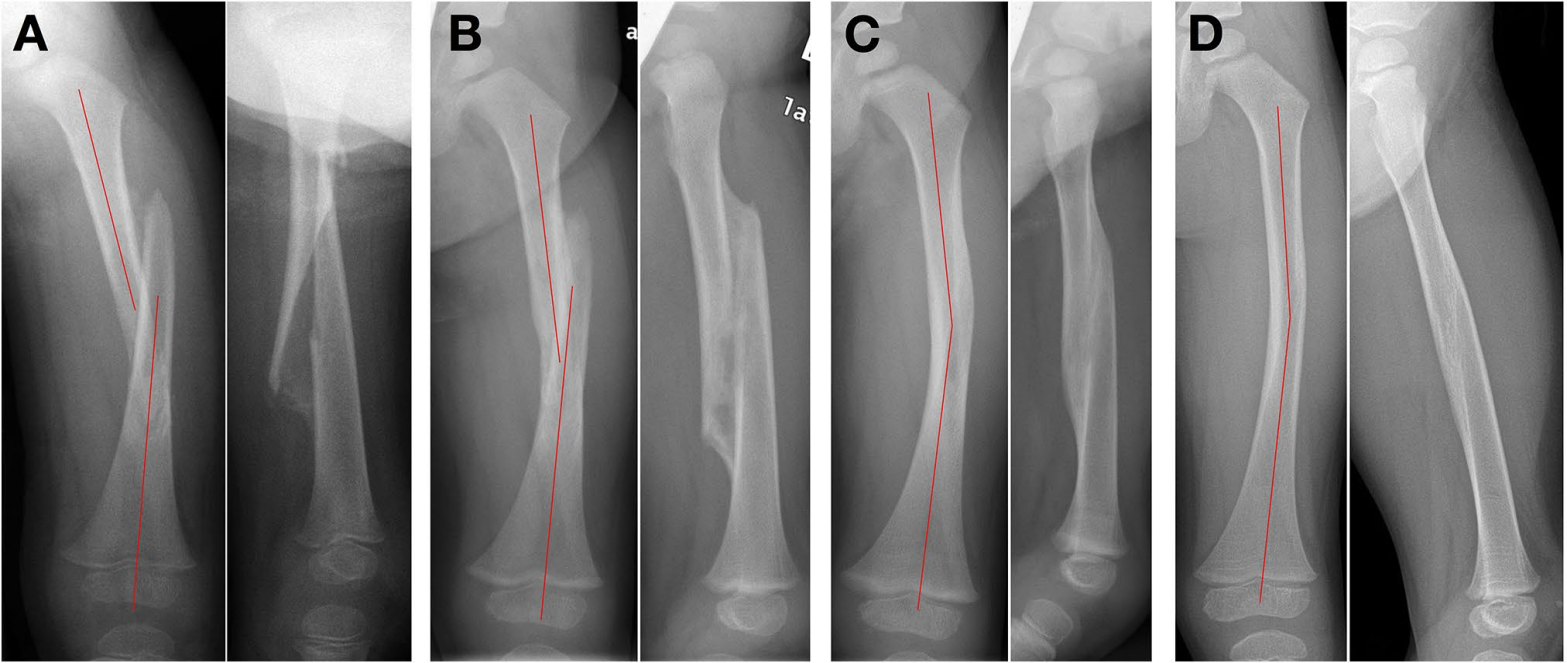
The remodeling of the femoral shaft after skin traction in a 2.5-year-old boy: consolidation after traction treatment (**A**) and controls after two months (**B**), six months (**C**), and 21 months (**D**)

**Fig. 3:**
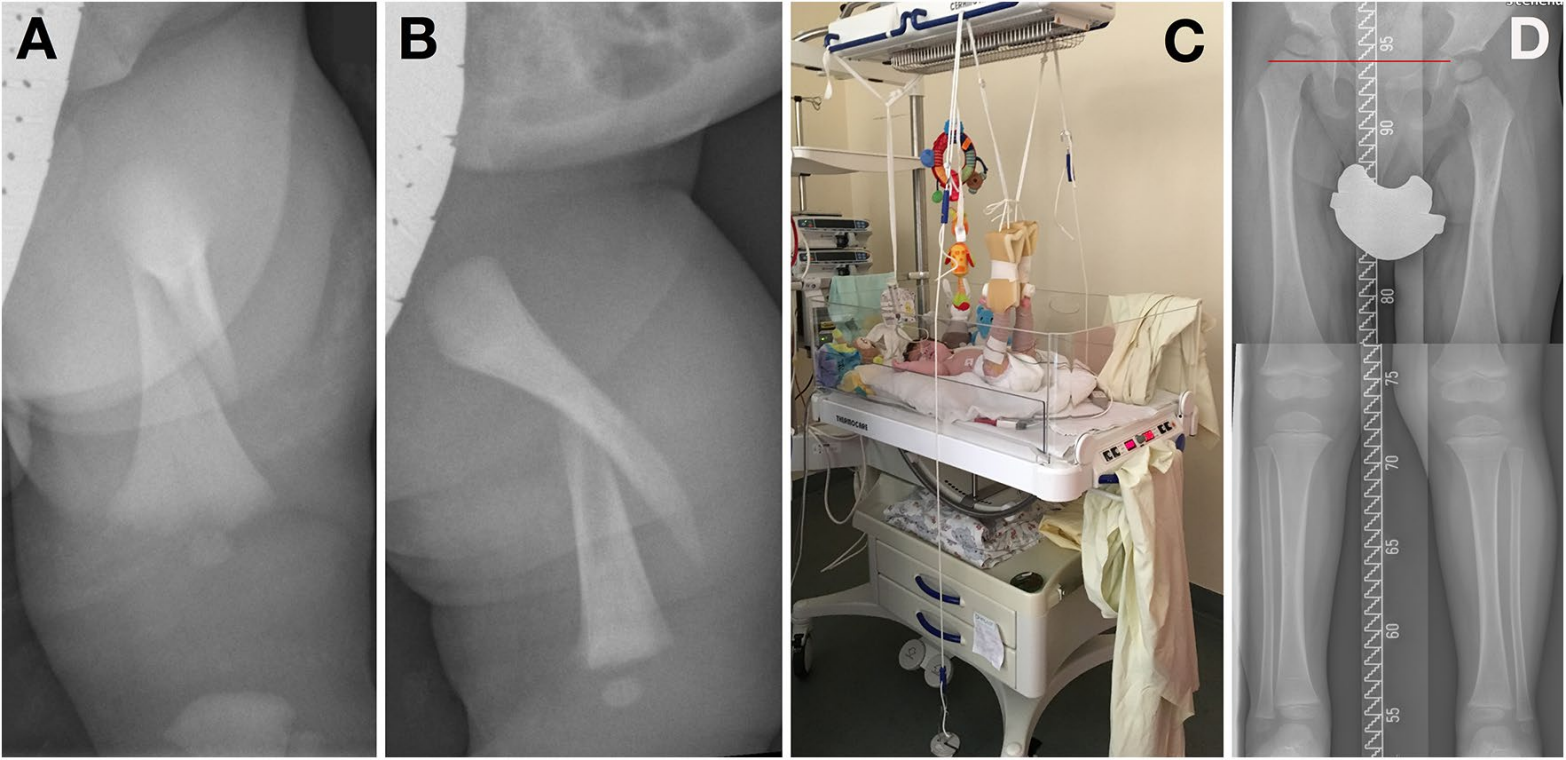
A birth-related femoral shaft fracture in a newborn: initial fracture (**A**, **B**), the newborn during skin traction (**C**), and radiographic outcome at the age of 2.5 years (**D**). The shortening of the fractured limb is 13 mm, but no restrictions on daily activities or limitations during sports have been reported

The complication rate in the present collective is 13.9%. However, only superficial skin erosions and blisters were documented. All of these issues healed spontaneously corresponding to a grade 1 within the classification proposed by Dindo/Clavien [27]. After ESIN, complication rates between 1.7 and 13.8% have been reported [28–30]. Here, an increase of complications has been found depending on higher bodyweight and/or older age [31], but no data for the group below four years of age are currently available. For skeletal traction, healing disorders at the Steinman pin have been reported at 9.4%, and operative sequestrectomy was indicated in some of the cases [32]. Nevertheless, significant complications have also been reported with skin traction [14, 24]. One example is a compartment syndrome with consecutive growth disturbance and shortening in the non-fractured limb because of ischemic arrest of all physes distal to the knee [23, 33]. Furthermore, axial deviations and leg-length discrepancies have been mentioned in all collectives. However, the results obtained undergo considerable modifications during further growth, particularly due to bone remodeling, the correction of angular deformities, and growth stimulations of the affected bone (Fig. 2). Several authors report a mean overgrowth of 6.9–13.0 mm at the affected limb due to biological stimulation of growth plates, caused by the hyperemia produced during consolidation and remodeling [34]. Corry et al. report age to be an influential variable, and overgrowth being less in children below four and those above seven years of age [35]. Stilli et al. found a greater overgrowth in all children below five years of age [36]. Also, overgrowth of the non-fractured ipsilateral tibia was observed in the case of femoral fractures [34]. However, when planning treatment of displaced femoral shaft fractures, an overriding (which should vary according to the age of the patient) up to 1.2 cm is suggested [37]. This has been well implemented in our collective and explains the results at the last follow-up. Considering that growth stimulation is persisting until 3.5 years after the fracture [38], even better results can be expected in those patients with short follow-up intervals. Remodeling of torsional deformities is seen much more critically in the literature; therefore, a safe correction by traction was carried out, although clinically these deformities are well compensated. Nevertheless, our data illustrate that skin traction is not an anatomically corrective therapy, but it allows rapid consolidation and good results through remodeling without the need for general anesthesia. This applies especially to birth-related femoral shaft fractures (Fig. 3). Once again, it must be stressed that the method itself does not include full correction of length and axis but relies on including the growth-related potential of correction as a treatment principle.

Even though good results with skin traction for femoral shaft fractures have been obtained, limitations of our study must be addressed. Essentially, these are its focus on a single center and retrospective characteristics. Furthermore, the period of follow-up is relatively short in some of the patients, and a comparison with alternative conservative and surgical methods has not been performed. However, this study analyzes a highly homogenous group of children below the age of four years. For this specific age group, skin traction as a treatment for femoral shaft fractures still seems justified for medical and socio-economic reasons. Here, the evaluation during medical rounds, communication with healthcare professionals, and care provided by the nurses is indispensable and significantly affects the contentment of the families [13]. To further address the age-specific treatment of femoral fractures in children, an observational Pediatric Femur Fracture Registry (PedFemFx) in Europe and Northern America has recently been completed in cooperation with AO international, and the first results are expected soon [39].

## Conclusion

Skin traction is a technically easy, safe, and non-invasive treatment modality for femoral shaft fractures in children below the age of four years. Strong results are obtained due to a high potential of growth-related correction, and in principle, no anesthesia is needed for this technique. A prolonged hospitalization and socio-economic factors maintain the ongoing debate in comparison with the surgical approach.

## Data Availability

The datasets analyzed during the current work are available upon reasonable request from the corresponding author.
